# Plasmonic metamaterial for electromagnetically induced transparency analogue and ultra-high figure of merit sensor

**DOI:** 10.1038/srep45210

**Published:** 2017-03-23

**Authors:** Dong Wu, Yumin Liu, Li Yu, Zhongyuan Yu, Lei Chen, Ruifang Li, Rui Ma, Chang Liu, Jinqiannan Zhang, Han Ye

**Affiliations:** 1 State Key Laboratory of Information Photonics and Optical Communications, Beijing University of Posts and Telecommunications, Beijing 100876, China

## Abstract

In this work, using finite-difference time-domain method, we propose and numerically demonstrate a novel way to achieve electromagnetically induced transparency (EIT) phenomenon in the reflection spectrum by stacking two different types of coupling effect among different elements of the designed metamaterial. Compared with the conventional EIT-like analogues coming from only one type of coupling effect between bright and dark meta-atoms on the same plane, to our knowledge the novel approach is the first to realize the optically active and precise control of the wavelength position of EIT-like phenomenon using optical metamaterials. An on-to-off dynamic control of the EIT-like phenomenon also can be achieved by changing the refractive index of the dielectric substrate via adjusting an optical pump pulse. Furthermore, in near infrared region, the metamaterial structure can be operated as an ultra-high resolution refractive index sensor with an ultra-high figure of merit (FOM) reaching 3200, which remarkably improve the FOM value of plasmonic refractive index sensors. The novel approach realizing EIT-like spectral shape with easy adjustment to the working wavelengths will open up new avenues for future research and practical application of active plasmonic switch, ultra-high resolution sensors and active slow-light devices.

In the past years, the classical analog of EIT-like phenomenon has attracted great research interest due to their many applications in slowing down light^1^,^2^,^3^,^4^, highly sensitive sensor^5^,^6^,^7^, quantum information processor^8^,^9^,^10^ and active plasmonic switch^11^,^12^. Originally, EIT phenomenon is realized in quantum interference effects that appear in three-level atomic systems by resulting in a sharp transmission window within a wide reflection or absorption band. Unfortunately, the quantum interference phenomenon is strictly limited by scathing environments in practical application, such as stable gas lasers and cryogenic temperature. Recently, analogues of the EIT-like phenomenon in plasmonic metamaterials have been designed and investigated under normal environment, which bring the quantum interference phenomenon into the classical systems. To date many researchers have realized the EIT-like phenomenon from various metamaterial structures for their attractable applications^13^,^14^,^15^,^16^,^17^,^18^,^19^,^20^. All previous studies about EIT-like effects based on metal plasmonic metamaterials is under the guideline of the destructive interference (or coupling effect) between bright and dark elements to form narrow transparent peak within the broader reflection or absorption spectrum^21^,^22^,^23^,^24^,^25^,^26^. For example, Na *et al*. provide the first experimental demonstration of EIT-like effects in optical metamaterials, comprising a gold bar stacked above two symmetric gold wires^19^. The gold bar acts as the bright element due to strong coupling of the incident light, whereas the gold wires act as the dark mode. Gu *et al*. demonstrate a EIT metamaterial consisting of a cut wire(CW) and two split-ring resonators (SRRs)^21^. The CW resonator and the SRR-pair serve as the bright and dark modes, respectively; Yang *et al*. present experimental observation of an EIT effect in a double-gap split-ring resonator. The rectangular bar resonator couples strongly to free-space excitation with the incident light, which form the bright mode resonance^13^. The ring resonators interact through near-field coupling, forming the dark mode of the system; Miyamaru *et al*. propose and demonstrate an optical switching based on an EIT-like spectral shape in the reflection spectrum, which consists of an array of complementary SRRs and rectangular slits deposited on a GaAs substrate^4^. Obviously, in these methods, the wavelength position of realizing EIT-like phenomenon strongly depends on the resonance wavelength of bright mode and dark mode, owing to their origins of the coupling effect. The resonance wavelength of bright and dark elements is hard to vary linearly with parameters of the structure simultaneously. Thus, for a metamaterial structure, the EIT-like phenomenon can only be achieved at the specific wavelength, which is difficult to extend to other frequencies nor to realize the precise control of wavelength position. For many applications, it is necessarily desirable to dynamically tune the EIT-like phenomenon to different frequencies. However, so far there is no report on EIT metamaterials based on plasmonic structure whose operating wavelength can be tuned dynamically in an approximate linear relation. It is complicated and bothersome to realize EIT-like phenomenon at different frequencies by designing various metamaterial structures using the conventional way. The operating wavelength conventional EIT-like analogues is not easy to extend and adjust, which greatly limits their further application and development.

Here, we propose a novel approach for realizing EIT-like phenomenon in the reflection spectrum at mid-infrared region by stacking two different types of reflection dips. The two reflection dips resulting from two different types of coupling effects between two gold nanobars and a gold thin film are respectively sensitive to the index of dielectric layer and substrate. The two reflection dips are partly overlapped in spectrum, which result in the EIT-like phenomenon. The optical control of the refractive index of some dielectric materials have been achieved in refs 4, 20, 27, 28, 29, 30, 31, 32, 33. Thus, the metamaterial structure can achieve the dynamic switching between on and off states by adjusting excitation power of optical pump pulse, owing to the dielectric layer/substrate using the light-sensitive material. Moreover, the wavelength position of the two reflection dips changes linearly with the refractive index of dielectric layer and substrate respectively. Then, the precise control of operating wavelength of EIT-like phenomenon also is achievable. The optical control is the most rapid regulation method in practical operation. Thus, compared with the conventional EIT-like analogues just using one type of coupling effect (destructive interference) between bright and dark elements, the new method can achieve EIT-like spectral shape with excellent regulation performance to the working wavelengths. To the best of our knowledge, the EIT-like phenomenon based on metal plasmonic metamaterials is firstly demonstrated in the mid-infrared region. Previously, the EIT phenomenon is mainly reported at near infrared (including telecom wavelength regime), THz and microwaves region in previous EIT metamaterials. To expand the operating regime, we also numerically demonstrate an optical switch based on this novel method with the operating wavelength around 1550 nm by scaling down the size of the unit cell. Furthermore, the designed structure can be worked as a refractive index sensor using the resonance dip resulting from surface plasmon polarizations (SPP) in near-infrared region. This plasmonic nanostructure shows an excellent sensing performance with an ultra-high FOM of 3200, which greatly improves the FOM value compared with previously reported refractive index sensor based on plasmonic metamaterials^34^,^35^,^36^,^37^,^38^,^39^,^40^,^41^,^42^,^43^,^44^,^45^,^46^,^47^,^48^,^49^. Although the improvement of FOM has already attracted considerable attention among many researchers, it is still a challenge to design a plasmonic refractive index sensor with an ultra-high FOM. For example, Kubo *et al*. experimentally demonstrate a plasmonic sensor based on Au double nanopillar (DNP) arrays, which has a high figure of merit reaching 24.4^46^. Yong *et al*. propose and numerically demonstrate an all-metal plasmonic refractive index sensor with a sensitivity of 885 nm/RIU and figure of merit as high as 110^34^. Lin *et al*. design and investigate a refractive index sensor with an ultra-high figure-of-merit (254), which is based on the Au bowtie nanoantenna arrays with metal-insulator-metal configuration^37^. Shen *et al*. describe and demonstrate a LSPR sensor with a FOM reaching108, consisting of an array of submicrometer gold mushrooms^42^. Recently, Jeong *et al*. demonstrate a refractive index sensor based on the localized surface plasmon resonance (LSPR) of individual metallic nanoparticles^49^. The plasmonic sensor has a refractive index sensitivity of 1091 nm/RIU and a FOM of 2800, which is the highest FOM proved by experiment at present. With the exception of the plasmonic sensor reported by Jeong, the most reported plasmonic refractive index sensors have a relatively low FOM < 300^34^,^35^,^36^,^37^,^38^,^39^,^40^,^41^,^42^,^43^,^44^,^45^,^46^,^47^,^48^.

## Results

### Coupling behavior in the designed metamaterial

**Figure 1:**
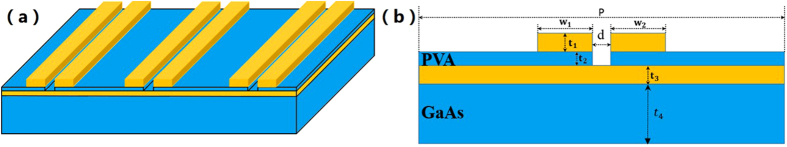
(**a**) Schematic of the proposed structure (**b**) Cross-section of the proposed structure in one unit. The geometrical parameters are: P = 2400 nm, w = w_1_ = w_2_ = 331 nm, d = 26 nm, t_1_ = 33 nm, t_2_ = 6 nm, t_3_ = 24 nm and t_4_ = 1.97 *μ*m.

**Figure 2:**
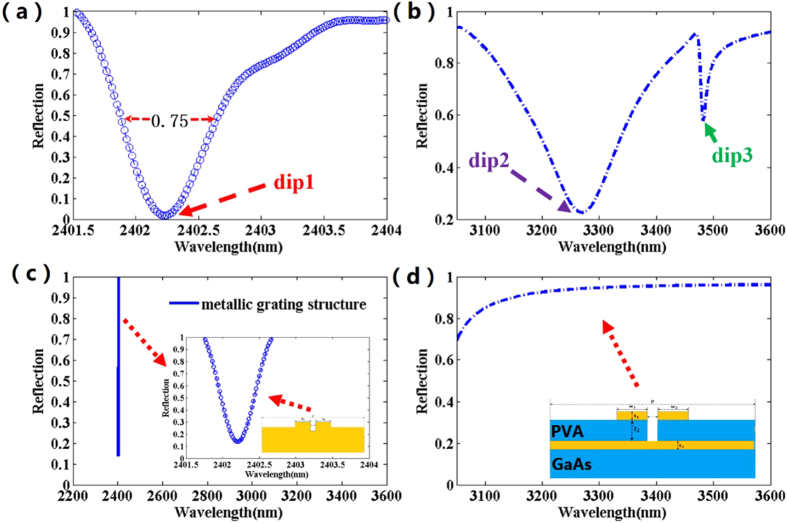
(**a**) The simulated reflectance spectrum of the proposed structure in the near-infrared region. (**b**) The simulated reflectance spectrum of the proposed structure in mid-infrared range. (**c**) The simulated reflectance spectrum of metallic grating structure. (**d**) The simulated reflectance spectrum of the structure with a large t_2_ = 500 nm, when the other parameters are fixed.

As shown in Fig. 1(a), the proposed EIT metamaterial consists of a periodic gold nanobars array and a gold film separated by a PVA (poly(vinyl alcohol), refractive index of n_PVA_ = 1.5) layer supported by a GaAs(n_GaAs_ = 3.55) substrate^4^,^20^,^27^,^28^,^29^,^30^,^31^,^32^,^33^. Figure 2(a) and (b) respectively present the reflection spectra of the proposed metamaterial structure in the near infrared and middle infrared spectral region. Figure 2(a) indicates that it is easy to observe the existence of a sharp reflection dip in the near-infrared range with a very low reflectivity of the resonance dip and an ultra-narrow bandwidth about 0.75 nm. As shown in Fig. 2(b), two reflection dips are obtained in mid-infrared region. For comparison, the reflection spectrum for the metallic grating structure with the same geometric parameters are calculated and presented in Fig. 2(c), which only has a sharp reflection dip at the wavelength of 2402.2 nm. However, unlike the original structure, there’s no reflection dips exist at the mid-infrared region in the reflection spectrum of the metallic grating structure. Thus, for the original structure, the two reflection dips in mid-infrared region might have close relationship with the coupling between the gold nanobars and the thin film. The physical mechanism for the sharp reflection dip at near infrared may be quite different from the mechanisms for the two reflection dips at mid-infrared region. To further research the structural effect of the gold nanobars and the film on the resonant dips in the same frequency region, Fig. 2(d) shows the reflectance spectrum of the structure having a large distance t_2_ between the gold nanobars and the film. Obviously, compared with the reflection spectrum of the original structure at Fig. 2(b), the reflection spectrum of the structure have no resonance effect in the mid-infrared range due to the lack of coupling between the nanobars and the film. Thus, both the dip2 and dip3 originate from the coupling effect between the nanobars and the gold film.

**Figure 3:**
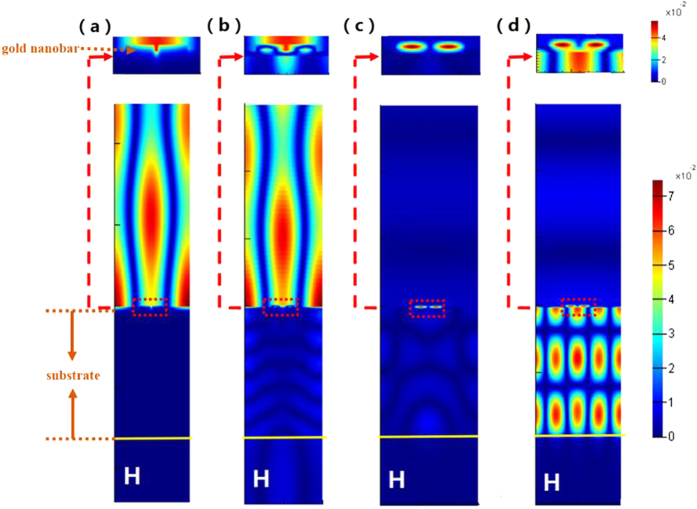
(**a**) The magnetic field distribution at the resonant wavelength of the metal grating structure. The magnetic field distribution at the (**b**) resonance dip1, (**c**) resonance dip2 and (**d**) resonance dip3 of the proposed structure.

In order to clarify the underlying mechanism of the these reflection dips, the magnetic field distributions at the resonant wavelength of the metal grating structure and the original structure are simulated and depicted in Fig. 3(a–d) respectively. As show in Fig. 3(a), the magnetic field is located on the surface of the grating-like gold structure which indicates that the surface plasmon polaritons (SPP) are excited by the incident waves coupled with the gold grating structure and the surface plasmons cannot pass through the metal and only propagate on the metal surface. Figure 3(b) shows that the magnetic field at the resonance dip1 is mainly distributed between the two gold nanobars and some magnetic field is located in the dielectric layer, which indicating that the mechanism of the resonance dip1 is same with that of the resonance dip of the metallic grating structure. Moreover, as shown in Fig. 3(c,d), the dip2 and dip3 obviously result from the coupling effect among the gold nanobars and the film, which can be indicated by the strong magnetic field located between the nanobars and the film. And the result is consistent with the comparative analysis between Fig. 2(b) and (d). Note that, the magnetic field distribution at the resonance dip2 is only distributed between the nanobars and the gold film, and the magnetic field distribution at the resonance dip3 is mainly located in the substrate and some magnetic field is distributed between the nanobars and the film. Thus, this may make the reflection dip2 and reflection dip3 very sensitive to the variation of the index of the PVA layer and the GaAs substrate respectively. The refractive index of the PVA and GaAs can be tunable by varying the excitation power of optical pump pulse^4^,^20^,^27^,^28^,^29^,^30^,^31^,^32^,^33^, which provide the possibility for the optical control of the stacking the two reflection dips.

### The optical and precise control of the EIT-like phenomenon

The metamaterials to realize EIT-like phenomena have been extensively studied in previous studies^13^,^14^,^15^,^16^,^17^,^18^,^19^,^20^. Particularly, it is desirable to achieve active control of EIT-like phenomenon in practical application and the optical tuning of EIT-like phenomenon has attracted more and more interest owing to its excellent performance in ultrafast optical switching and ultrafast modulation speed. However, the conventional EIT analogues result from the destructive interference between the bright mode and dark mode, and the dark mode is excited by the near field coupling between bright and dark meta-atoms. Obviously, the dark mode is strongly associated with the bright mode. Thus, it is difficult to change the properties of bright mode and dark mode independently. These disadvantages greatly affect the flexibility of controlling the EIT-like phenomenon in practical application. Different from the conventional way to realize EIT metamaterials, the EIT-like phenomenon in this work originate from stacking two different types of coupling effect in the structure, which can be tuned independently and actively.

**Figure 4:**
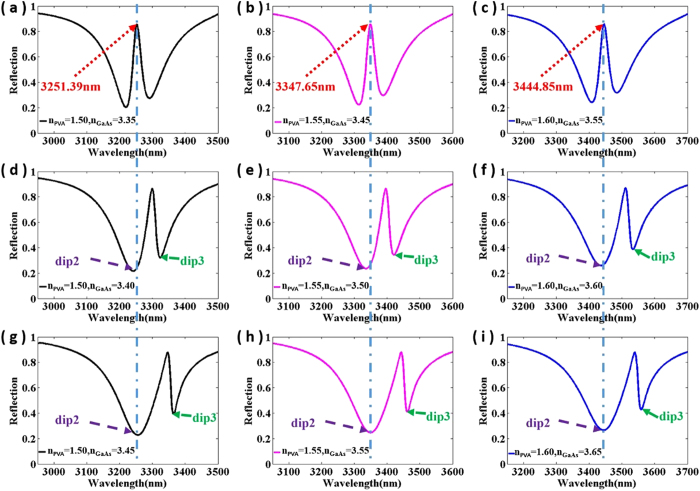
The reflection spectra of the structure with the different refractive index of PVA layer and GaAs substrate.

As shown in Fig. 4, the reflected spectra of the metamaterials with slight changes in refractive index of PVA layer and GaAs substrate are calculated and analyzed. When n_PVA_ = 1.5 and n_GaAs_ = 3.35, it is obviously observed that a narrow reflection peak exits at 3251.39 nm within a wide refection band ranging from 2950 nm to 3500 nm (shown in Fig. 4(a)), which is a typical feature of EIT-like effect. From Fig. 4(a,d,g), it is noticeable that the refractive index variation of GaAs substrate can bring about switching between on and off states at 3251.39 nm, as the dip 3 is sensitive to the index of GaAs substrate but the dip 2 is independent, which is in good agreement with the results of magnetic field distributions in Fig. 3(c,d). Similarly, comparing the illustrations in Fig. 4(b,e,h) or Fig. 4(c,f,i) respectively, it is observed that the metamaterial structure can also achieve the switching between on and off states in the wavelength of 3347.5 nm or 3444.85 nm by slightly changing the refractive index of the GaAs substrate. Then, as shown in Fig. 4(a–c), it is obvious that the operating wavelength realizing EIT-like phenomenon can be easily adjusted by slightly changing the refractive index of PVA layer and GaAs substrate, which can be well explained by the magnetic field distributions in Fig. 3(c,d). Thus, the designed metamaterial can realize the active and precise control of the position of EIT phenomenon by slightly changing the index of the PVA layer and GaAs substrate. The refractive index of GaAs/PVA can be easily changed by pump light^4^,^20^,^27^. Because the carrier lifetime in dielectric generally is tens of picosecond, the vast majority of photo-excited carriers still exist during the illumination of incident light. Thus, the dielectric constant in the dielectric layer or substrate can be consider to be constant during this time. Then, the change in refractive index of the dielectric layer is induced by photo-excited carriers, which can be produced by the various excitation power of pump light. Finally, the pump light can change the reflection dips to dynamically realize all-optical switches and precise control of EIT-like phenomena, since the reflection properties of the metamaterial are extremely sensitive to the refractive index of dielectric layer and substrate.

**Figure 5:**
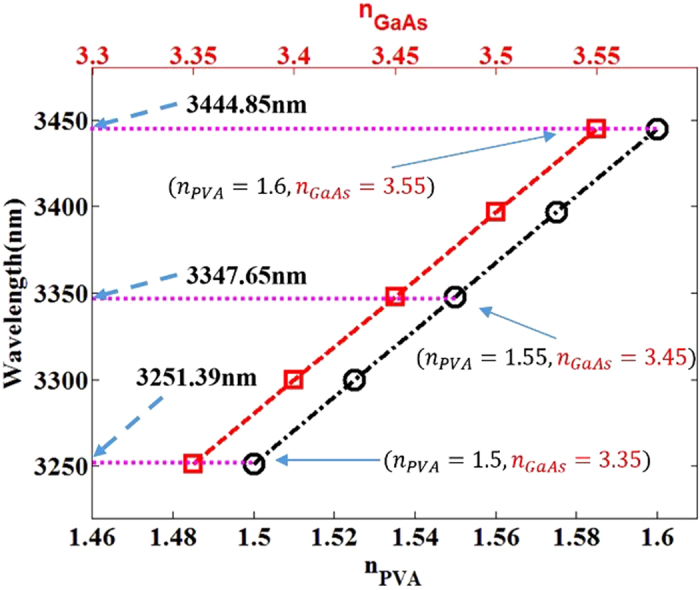
The relationship between operating wavelength of the EIT metamaterial and the refractive index group in PVA and GaAs.

In order to describe more accurately the relationship between the refractive index of dielectric layer/substrate and the operating wavelength of EIT-like phenomena of proposed structure, Fig. 5 presents the operating wavelength of the metamaterial to achieve EIT-like effect with the variation of the refractive index in PVA/GaAs. Obviously, the operating wavelength has a linear relation with the combination of refractive index in the PVA and GaAs. It means that a wide range of operating wavelength for the EIT-like phenomenon can be precisely and dynamically achieved, by varying the refractive index in the PVA/GaAs layer induced by photo-excited carriers.

**Figure 6:**
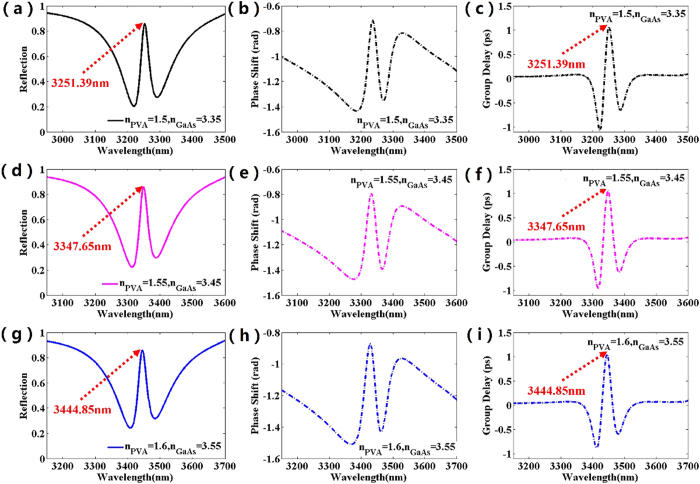
The reflection spectra, phase shift and group delay at different operating wavelength for the different refractive index of PVA layer and GaAs substrate.

Next, as shown in Fig. 6(b,e,h), the phase shift spectra of reflected waves from the metamaterial with different refractive indexs of PVA/GaAs is investigated. Generally, the phase shift spectra is obtained by *φ*_sam − _*φ*_metal_. The *φ*_sam_ is the phase spectrum of the reflected wave from the proposed metamaterial sample and *φ*_metal_ is that from a metal mirror, which replace the metamaterial sample as reference material. As shown in Fig. 6(b,e,h), it is easily observed that a linear and steep gradient of the phase shift slope exists around 3251.39 nm, 3347.65 nm, 3444.85 nm respectively. Then, the group delay spectra can be calculated through the gradient operation to these phase shift spectra. Obviously, from Fig. 6(c,f,i), the group delay of reflected waves is demonstrated at different wavelengths, which result from the slight variations in refractive index of dielectric. A group delay above 1 ps can be achieved at 3251.39 nm, 3347.65 nm and 3444.85 nm, respectively. With increasing the index of PVA from 1.5 to 1.6, the group delay spectra shifts obviously to higher wavelengths owing to the sensitivity of EIT-like phenomenon to refractive index. Note that the index of PVA can be changed dynamically by pump light^27^,^28^,^29^,^30^,^31^,^32^,^33^, indicating that the metamaterial structure realizing the group delay of reflected wave can actively and dynamically operate at different frequencies.

**Figure 7:**
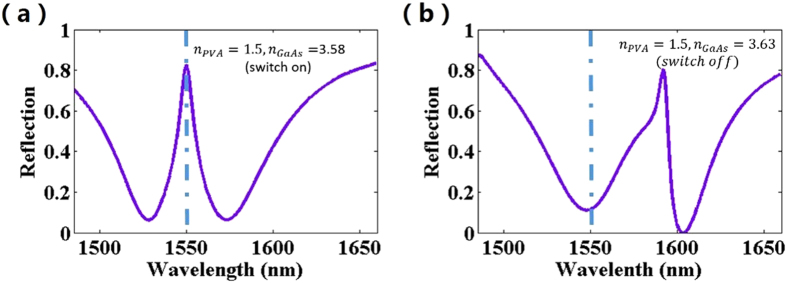
The simulated reflectance spectra that reveal the on and off states at 1550 nm. The geometrical parameters are P = 1050 nm, w = w_1_ = w_2_ = 198 nm, d = 65 nm, t_1_ = 30 nm, t_2_ = 12 nm, t_3_ = 26 nm and t_4_ = 2.682 *μ*m.

In order to expand the operating region, as shown in Fig. 7, we also numerically demonstrate the metamaterial structure to realize the EIT-like phenomenon at the wavelength of 1550 nm by scaling down the size of the unit cell. Figure 7 depicts a plasmonic switch at 1550 nm, where the broad reflection dip with a sharp reflection peak at 1550 nm is switched on at Fig. 7(a) and that of the metamaterial is switched off at Fig. 7(b). Thus, the metamaterial structure can easily achieve the all-optical plasmonic switches in other frequency bands by changing the size of the unit cell, which provides a convenient feature to design slow light devices different frequency ranges.

### Ultra-high FOM plasmonic refractive index sensors

**Figure 8:**
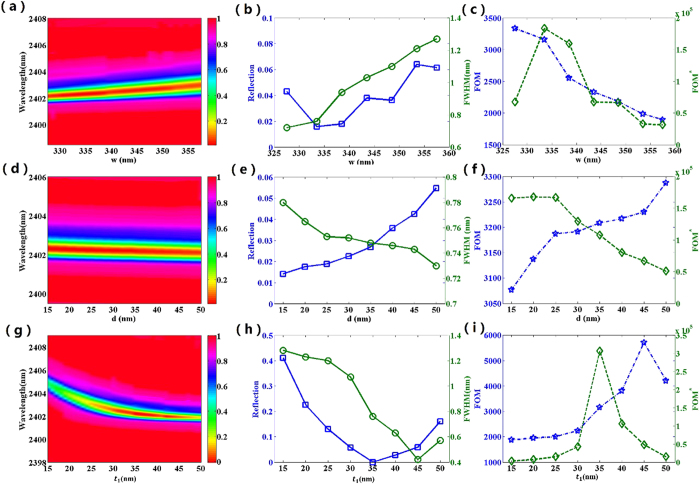
The dependence of reflectance spectrum of the proposed structure on (**a**) nanobar width w, (**d**) the distance between nanobars d and (**g**) nanobar thickness t_1_. The FWHM and reflectivity dip as functions of (**b**) nanobar width w, (**e**) the distance between nanobars d and (**h**) nanobar thickness t_1_. FOM and FOM* as functions of (**c**) nanobar width w, (**f**) the distance between nanobars d and (**i**) nanobar thickness t1.

In recent years, plasmonic metamaterial has attracted much attention due to its various potential applications, especially in the sensing field^34^,^35^,^36^,^37^,^38^,^39^,^40^,^41^,^42^,^43^,^44^,^45^,^46^,^47^,^48^,^49^. The FOM is the key parameter for evaluating the sensing performance of plasmonic sensor. For example, in the biological area, it is well known that the higher FOM means the plasmonic refractive index sensor with a better performance to detect molecule. However, the most reported plasmonic refractive index sensors have a relatively low FOM <300 and it is still a challenge to design a plasmonic sensor with ultra-high FOM. Now we demonstrate that our metamaterial structure can operate as an ultra-high FOM refractive index sensor in the near infrared region. With the variation of the surrounding refractive index, the sensitivity(S) can reach 2400 nm/RIU, which have been demonstrated based on a similar plasmonic structure in our previous study^50^. The full width at half maximum (FWHM) of the reflection dip1 reaching 0.75 nm at Fig. 2(a), This FWHM is much narrower than those of MDM based perfect absorbers, whose FWHM are 3.94 nm, 7.5 nm and 9.5 nm in refs 34, 37 and 42, respectively. Thus, the FOM = S/FWHM = 3200 is obtained. Furthermore, in order to further evaluate the sensing performance of the plasmonic structure, we also investigate the optical performance and the sensing performance of the plasmonic refractive index sensor dependence on different structure parameters, as shown in Fig. 8. It is well known that the narrower FWHM of the reflection dip and the lower value of the resonance dip are essential for refractive index sensor. By adjusting the structure parameters, a better value of reflection dip or FWHM can be achieved. As shown in Fig. 8(b,e,h), the optimized value of FWHM and reflectivity of the resonance dip cannot be obtained simultaneously. However, obviously, in our design, the value of reflection dip remains low and meanwhile FWHM changes slightly in a wide range of structure parameters variation, which is favorable for designing refractive index sensor to practical application.

In practical application, an intensity change of reflected wave can be detected at a fixed wavelength, which result from the change of surrounding refractive index. The corresponding figure of merit is defined as 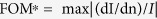, where 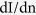 is the relative intensity change owing to the refractive index change and I corresponds to the light intensity at the fixed wavelength. As shown in Fig. 8(c), when w increase from 325 nm to 360 nm, the plasmonic sensor shows decrease of FOM and a maximum of FOM*. From Fig. 8(f), the structure presents increase of FOM and decrease of FOM* as d increases. As shown in Fig. 8(i), the maximum value of FOM and FOM* can respectively reach up to 5700 and 3 × 10^5^, which is higher than those of most previously reported refractive index sensor based on plasmonic metamaterial^34^,^35^,^36^,^37^,^38^,^39^,^40^,^41^,^42^,^43^,^44^,^45^,^46^,^47^,^48^,^49^. Due to the ultra-high FOM and FOM*, the plasmonic refractive index sensor has great potential in biomedical and chemical applications.

### Perfect ultra-narrow band absorber

**Figure 9:**
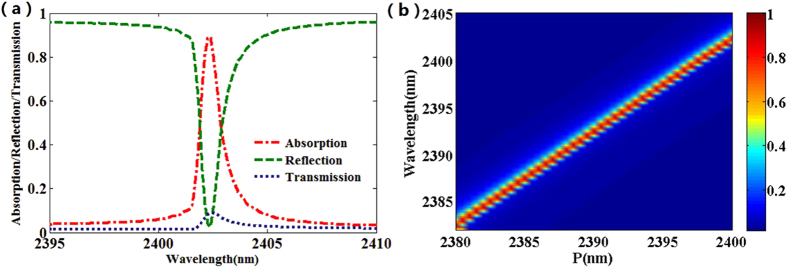
(**a**) Simulated reflection, transmission and absorption spectra of the proposed structure. (**b**) The absorption spectra of the proposed structure as a function of the periods P.

The narrow band absorbers based on plasmonic nanostructures have various applications in thermal emitters, optical filters and sensors. However, it is still a challenge to design plasmonic absorbers with ultra-narrow band, high absorption and a wide range of operating wavelength. Here, the metamaterial structure also can be operated as an ultra-narrow band near perfect absorber in near-infrared region. As shown in Fig. 9(a), there is a high absorption with an absorption reaching 90% and an ultra-narrow band of 0.75 nm. Figure 9(b) presents the absorption spectra of the ultra-narrow band absorber as a function of the periods of structures. Obviously, the absorber can obtain a wide operating wavelength by change the size of the structure in on unit cell.

## Discussion

In summary, the proposed metamaterial structure successfully realize the EIT-like phenomenon by using our novel way of stacking two different types of coupling effect. Compared with conventional method, this novel approach is demonstrated firstly to realize the active and precise control of EIT-like phenomenon by slightly changing the refractive index of dielectric. As the refractive index of GaAs/PVA can be easily changed by pump light, it is noticeable that the EIT-like phenomenon can be optically controlled. An active and optical switch of the EIT-like phenomenon is also numerically demonstrated. The proposed metamaterial structure can be easily applied to different frequency ranges by scaling the structure sizes. Moreover, by means of numerical simulation, the metamaterial structure can operate as an ultra-high resolution refractive index sensor with an ultra-high FOM reaching 3200. The proposed novel metamaterial will easily find practical applications in active plasmonic switch, ultra-high resolution sensors and active slow-light devices.

## Methods

The two-dimensional finite-difference time-domain (FDTD) method was employed to analyze the optical performance of the proposed nanostructure. In the simulations, the permittivity of the gold was characterized by the Drude model. A TM wave is normally incident onto the plasmonic nanostructure along the -z direction, with its electric field E along the x direction. Periodic boundary conditions are applied at the lateral boundaries, and perfectly matching layers are employed along the z direction. The mesh size is set to be 0.1 nm which is much smaller than the element sizes.

## Additional Information

**How to cite this article:** Wu, D. *et al*. Plasmonic metamaterial for electromagnetically induced transparency analogue and ultra-high figure of merit sensor. *Sci. Rep.* **7**, 45210; doi: 10.1038/srep45210 (2017).

**Publisher's note:** Springer Nature remains neutral with regard to jurisdictional claims in published maps and institutional affiliations.
